# Time-Varying Light Exposure in Chronobiology and Sleep Research Experiments

**DOI:** 10.3389/fneur.2021.654158

**Published:** 2021-07-15

**Authors:** Manuel Spitschan

**Affiliations:** ^1^Department of Experimental Psychology, University of Oxford, Oxford, United Kingdom; ^2^Centre for Chronobiology, Psychiatric Hospital of the University of Basel (UPK), Basel, Switzerland; ^3^Transfaculty Research Platform Molecular and Cognitive Neurosciences, University of Basel, Basel, Switzerland

**Keywords:** time-varying light exposure, circadian photoreception, non-visual effects of light, temporal stimuli, ipRGCs, melanopsin, reporting

## Abstract

Light exposure profoundly affects human physiology and behavior through circadian and neuroendocrine photoreception primarily through the melanopsin-containing intrinsically photosensitive retinal ganglion cells. Recent research has explored the possibility of using temporally patterned stimuli to manipulate circadian and neuroendocrine responses to light. This mini-review, geared to chronobiologists, sleep researchers, and scientists in adjacent disciplines, has two objectives: (1) introduce basic concepts in time-varying stimuli and (2) provide a checklist-based set of recommendations for documenting time-varying light exposures based on current best practices and standards.

## Introduction

Light profoundly affects human circadian and neuroendocrine physiology. Signals processed by the photoreceptors in the retina encode different aspects of environmental light. There are three classes of photoreceptors: the cones (of which there are three different spectral classes, the L, M, and S cones), the rods, and the melanopsin-containing intrinsically photosensitive retinal ganglion cells (ipRGCs). The retinal photoreceptors are sensitive to different but overlapping wavelength ranges, with melanopsin playing the primary role in mediating the circadian and neuroendocrine effects of light (1). Furthermore, the different photoreceptor classes also differ in the way they respond to light stimuli that are patterned in time, such as a train of brief flashes or sinusoidal flicker. With recent studies showing that flashes of light lead to different effects on circadian and neuroendocrine physiology than continuous light exposure (2, 3), it is worth reviewing how light stimuli changing over time can be described parametrically.

This tutorial paper is targeted to chronobiologists, sleep researchers and scientists from adjacent disciplines, such as environmental psychology, who wish to develop an understanding of specifying light exposure in time. The focus will be on how time-varying light stimuli can be described quantitatively in the time and frequency domain, learn about major classes of time-varying stimuli and their properties, and some caveats in using time-varying stimuli. This paper is providing an introduction to readers with no specific background in signal processing and analyses of time-varying signals.

## Parametric Descriptions of Time-Varying Stimuli

### Basic Concepts

#### Time-Domain Representation

**Figure 1:**
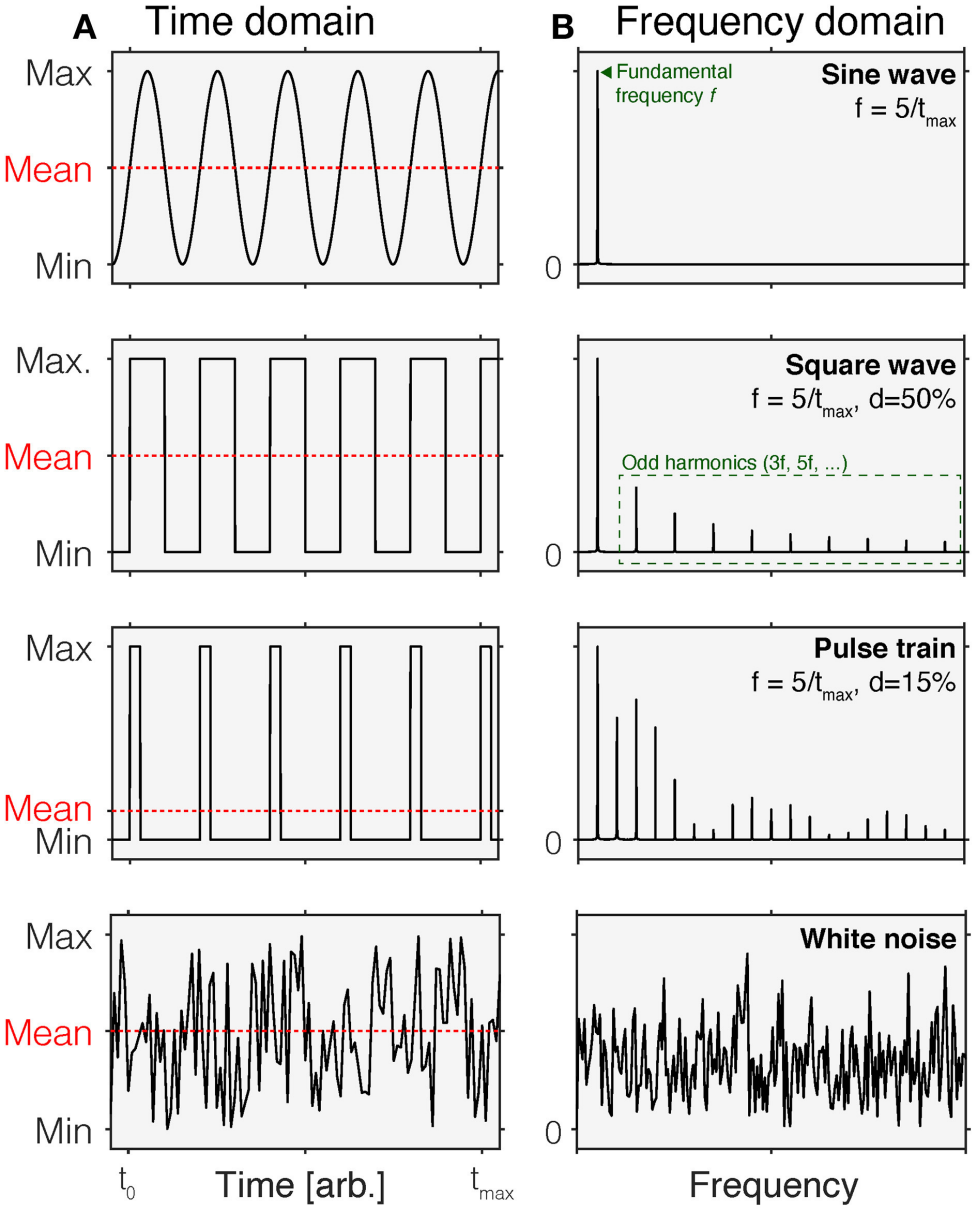
Time-domain **(A)** and normalized frequency **(B)** representations of common time-varying stimuli. In each panel in **(A)**, the mean of each waveform is indicated by the dashed red line. Different waveforms in the time domain have different properties in the frequency domain. *Row 1*: A sine wave is a periodic waveform going between minimum and maximum values. It only contains frequency components at the fundamental frequency *f* . *Row 2*: A square wave varies in a discrete fashion between minimum and maximum values. It is composed of sine waves of different frequencies, including the fundamental frequency *f* and odd harmonics (3f, 5f,…). In a square wave, the duty cycle is 50%, which means that over time, the fraction of time at the maximum value is 50% within a period. *Row 3*: A pulse train can be thought of as a square wave with duty cycles that are <50%. This shorting of the time of that the wave is at maximum introduces additional harmonics. *Row 4*: White noise corresponds to a signal that is sampled from a uniform distribution. In the frequency domain, this corresponds to a flat curve, indicating that all frequencies are equally represented.

An intuitive way of thinking about time-varying stimuli is by representing their variation as a function of time, i.e., as a time course (Figure 1A). This representation is called the time domain. In this representation, stimulus values vary across different time points. In most cases, this representation is discrete, i.e., there is a set of time points for which stimulus values are specified. These stimulus values could be the luminance or illuminance, or some variant of radiance or illuminance, depending on the space in which the stimuli are specified. They could also be specified in terms of the contrast relative to an explicit or implicit reference light, such as an adaptation light.

#### Frequency-Domain Representation

An equivalent, but to some perhaps less intuitive way of representing time-varying stimuli is the frequency-domain representation (Figure 1B). While the time-domain representation shows the change of a signal over time, the frequency-domain representation describes the extent to which a given signal happens at different frequencies. For example, a sinusoidal change in intensity with a period length of 1 s has a frequency of 1 Hz but has no power at any other frequencies. The power a given signal has at all frequencies (within a given range) is called the power spectrum. Time-domain and frequency-domain representations can be converted between each other using the Fourier Transform, which decomposes the periodic signal into its constituent, single-frequency components (4).

### Stimulus Spaces

The previous discussion mentioned two general classes of specifying waveforms: intensity and contrast. It is worth considering what actual quantities might be used for the specification of temporal waveforms and which units these might be expressed in. Here, we will review different ways of specifying the temporal waveform, focusing on absolute (intensity) and relative (contrast) units.

#### Intensity

The most basic representation of time-varying stimuli is a time course of spectral radiance or irradiance distributions, giving the radiance or irradiance of the stimulus at each wavelength for each point in time. At one point in time, this corresponds to a physical measurement (5) with no direct reference to the human photoreceptors. Spectra are weighted by the spectral sensitivities of the different photoreceptor classes (6), and then summed to arrive at alpha-opic radiance (for spectral radiance measurements) or alpha-opic irradiance (for spectral irradiance measurements). Box 1 provides a glossary of relevant terms. This can be accomplished using an Excel-based toolbox provided by the CIE (7, 8) and the recently published platform-independent open-access and open-source web software *luox* (https://luox.app/) (9).

#### Contrast

In cases where there is a well-defined background light around which stimuli are presented, e.g., sinusoidally flickering lights against a mean background, it is useful to specify the contrast of the flickering stimuli as the relative change in (il)luminance or alpha-opic (ir)radiance relative to the background (which is the mean of the signal for sinusoidal flicker). In certain cases, time-varying contrast could be specified in a cone-opponent space (10). This is essential in cases where the photoreceptor excitation is actively controlled, such as using metameric lights changing over time, or stimuli generated using the method of silent substitution (11). In the method of silent substitution, lights are designed in such a way that they only produce differences in the activation of a selected class of photoreceptors, with no difference in the activation of the other photoreceptor classes, which are “silent” to the exchange of lights.

#### Other Stimulus Spaces

It is important to note that time-varying stimuli defined in one stimulus space may not be linear when represented in another stimulus space. One such example is the mapping between correlated color temperature (CCT), a common way to specify the color appearance of lights relative to reference spectra (blackbody, <5,000 K or daylight spectrum, >5,000 K). A specification of CCT varying over time does not linearly map onto cone contrast changing over time, as CCT and cone activations are not linearly correlated. Consequently, such a non-linearity may lead to undesired frequency components in another space.

Box 1 **Glossary.**

**Radiance-derived quantities**

**Spectral radiance:** The spectral radiance is the absolute spectrum of light of a surface (self-emitting, e.g., displays, or reflecting, light reflected from a wall) per united solid angle per unit projected area. Spectral radiance is measured in W/m^2^/sr/nm. From the radiance, we can calculate various quantities:

- **Luminance** (cd/m^2^): Spectrum weighted by the photopic luminosity function.
- **α-opic radiances** (W/m^2^/sr): Spectrum weighted by the five α-opic effect functions corresponding to the spectral sensitivities of the human photoreceptors and summed. As a consequence, there are α-opic radiances (the letter “α” is just a placeholder). The **melanopic radiance** (W/m^2^/sr) is the spectrum weighted by the melanopsin spectral sensitivity function and summed.
- **α-opic equivalent daylight luminance (EDL)** (cd/m^2^): Luminance of a D65 daylight spectrum that produces the same α-opic radiance is the spectrum in question. The **melanopic equivalent daylight luminance (mEDL)** is in reference to the melanopic radiance.

**Irradiance-derived quantities**

**Spectral irradiance:** The spectral radiance is the absolute spectrum of light of a received by a given area. Spectral irradiance is measured in W/m^2^/nm. From the irradiance, we can calculate various quantities:

- **Illuminance** (lux): Spectrum weighted by the photopic luminosity function.
- **α-opic irradiances** (W/m^2^): Spectrum weighted by the five α-opic effect functions corresponding to the spectral sensitivities of the human photoreceptors and summed. As a consequence, there are α-opic radiances (the letter “α” is just a placeholder). The **melanopic irradiance** (W/m^2^) is the spectrum weighted by the melanopsin spectral sensitivity function and summed.
- **α-opic equivalent daylight luminance (EDL)** (lux): Illuminance of a D65 daylight spectrum that produces the same α-opic radiance is the spectrum in question. The **melanopic equivalent daylight illuminance (mEDI)** is in reference to the melanopic irradiance.

**Chromaticity** is an (il)luminance-independent way of specifying the color of an object, surface, or spectrum.

**Correlated color temperature (CCT)** is the temperature of a black-body radiator that matches the chromaticity of a spectrum in question.

**Contrast** is the relative different of activation (e.g., melanopic irradiance) between two different spectra.

## Basic Waveforms

### Non-periodic Waveforms

#### Pulses

A pulse is a change in stimulus intensity or contrast that has a limited duration. For example, a brief light flash in an otherwise dim environment is a pulse. When the intensity is pulsed around a background light, the more intuitive way to specify the stimulus properties is in terms of contrast, i.e., the relative difference in stimulus intensity with respect to the background light.

#### Ramps

Ramps are increases or decreases in intensity or contrast up or down to a specified level. An example of such a stimulus is a gradual increase of light intensity in “dawn simulation” lights (12–19). The term ramp itself is ill-defined, as ramps can be linear, exponential, logarithmic, or modulated in some other way, e.g., using a cosine window [e.g., (20)]. Different ramps have different parameters in the frequency space. Ramps are often used to remove transient signals at the onset of a light, smoothing out the abrupt transition between different spectra.

### Periodic Waveforms

#### Square-Wave Flicker

Square-wave flicker changes between two different intensity or contrast settings at a given frequency. A simple intuitive example is turning on the light switch in a room for 1 s, and turning it off again for 1 s.

#### Sinusoidal Flicker

Sinusoidal flicker is a light that is gradually changing intensity or contrast in accordance to the sine function. Sinusoidal flicker is parameterized by the frequency, phase, and the amplitude. In the frequency domain, sinusoidal flicker has the property that it only contains power at one frequency, the fundamental frequency. Square-wave flicker has power at odd harmonics as well.

#### Trains of Pulses

A sequence or train of pulses can be parameterized by the ratio between the duration that the light is on and the duration that the light is off. This is called the duty cycle and expresses the percentage that a light is turned on as part of the entire period. A duty cycle of 0% is of course simply no light, and a duty cycle of 100% is a continuous light. A repeating sequence of pulses with 50% duty cycle corresponds to the special case of square wave flicker (see above).

#### Other Waveforms

Of course, the space of possible waveforms is infinite. However, in studies employing stimuli parametrically, the waveforms above describe most use cases. Time courses sampled from different underlying distributions are called colored noise. For example, white noise (Figure 1, bottom row) has a flat frequency spectrum.

### Descriptors for Complex Time-Varying Stimuli in Their Experimental Context

Documenting and reporting of lighting conditions is key to ensuring that studies can be reproduced, aggregated, and placed into context. While guidelines detailing how static aspects of light should be documented have been developed recently (5, 21) and are worth consulting for details on spectral characterization of light, technical details on time-varying light exposure are currently not typically documented and reported in standard form. Recently, the International Commission on Illumination (CIE) published technical note CIE TN 011:2020 (6), describing which information should be captured in studies investigating non-visual responses to light. The document lists the following primary aspects that should be captured and documented: (1) the timeline of the experiment explained in detail, in clock time; (2) the duration of exposure in minutes or hours; (3) the sequence of exposures, including pre-experimental light and environmental exposures to the greatest degree of detail possible. The document expands this description to include (1) the overall duration of the experiment, (2) timing of light exposure (in clock time), (3) dim light exposure, (4) duration of the light exposure (in min or h), (5) duration of dim up and dim down (in min), and (6) pattern.

In an earlier document, CIE 213:2014 (22), the CIE suggested the following descriptors for dynamic light: (1) initial conditions (luminance, light source color, direction, etc.), specified as above; (2) intermediate and ending conditions, specified as above; (3) rate of change; (4) rate of cycle, if any; (5) change profile, particularly if the change is not linear; (6) movement of the observer's head and eyes relative to the light source or illuminated area. It is suggested to consult the two CIE documents already in the design stage of a research project.

In Table 1, we propose a reporting workflow based on the recommendations of CIE TN 011:2020 (6) and CIE 213:2014 (22) specifically for experiments in chronobiology and sleep research. In addition to naming specific quantities of interest and their derivatives, the proposed workflow also includes guidance on whether a specific item should be essential, optional, or recommended. The workflow is expandable and versatile. While it may be possible to derive this information from the full text of a given published study, future work should consider the development of a formalized schema which facilitates automated analyses based on stimulus descriptions as well as appropriate software tools that make it easy for investigators to provide this information. This may help make research future-proof (23) and more sustainable.

**Table 1:** Recommended reporting of time-varying stimuli.

**Aspect**	**Form**	**Status**
**Timeline of experimental protocol, including exposure durations and sequence of exposures**		
* Description of protocol timeline	Description in manuscript text	Essential
* Visualization of protocol timeline	Schematic visualization included as figure in main manuscript or supplementary figure	Optional, but recommended in particular for multiple-hour experiments
* Machine-readable tabular representation of protocol timeline	Data table included as supplementary material	Optional
**Static spectral measurements of all discrete light scenarios**		
* Spectral (ir)radiance distribution	Data table included as supplementary material	Essential
* CIE S026 quantities calculated from spectral measurements	Description in manuscript text or table in main text	Essential
OR		
**Static CIE S026 referenced measurements of all discrete light scenarios**		
* CIE S026 quantities	Description in manuscript text or table in main text	Essential
**Time series of time-varying stimuli and exposures specified as spectral or melanopic (ir)radiance [including pre-experimental light and**		
**environmental exposures** **(****6****)]**		
* Tabulated time series	Data table included as supplementary material	Essential
* Visualization of time series	Graph included as figure in main manuscript or supplementary figure	Optional, but recommended
* Representation of power spectra	Graph included as figure in main manuscript or supplementary figure	Optional
**Behavioral demands**		
* Instructions to participants regarding fixation and gaze direction	Description in manuscript text	Essential
* Verbatim text used to instruct participants	Description in manuscript text	Optional
**Pupil size**		
* Recording status (whether or not it was recorded)	Description in manuscript text	Essential
* Recording meta-data, including device details, sampling frequency, binocular or monocular recording	Description in manuscript text	Optional
* Mean pupil size per condition and statistical test showing whether they are different	Description in manuscript text	Optional
* Time series of pupil size data	Data table included as supplementary material	Optional

*Expanded recommendations and checklist based on CIE documents (6, 22)*.

* refers to derivatives. Essential reporting items are shaded in yellow.

## Considerations for Using Time-Varying Stimuli

### Non-linearities in Light Output at Different Driving Inputs

Light sources are generally controlled using an input parameter, which we will call the input settings. In a conventional 8-bit RGB monitor, for example, three primary lights can be controlled at 255 levels. However, the output radiance is not linear with the input RGB settings but instead follows a non-linear gamma function. As a consequence, for example, an RGB triplet of [127 127 127] does not represent the expected 50% of the maximum output radiance, but less. To be able to control a monitor linearly then requires a measurement of the gamma function. The same of course applies to other light sources.

Unless they are corrected, non-linearities in output radiance lead to distortions in the output signal. Consider, for example, the case of a sinusoidal modulation that is displayed using a non-linear light source. In the time domain, the resulting waveform is no longer symmetric around the mean. In the frequency domain, it becomes obvious that the non-linearities have introduced additional frequencies that were not present in the target modulation.

In addition to non-linearities in output radiance at different input settings, light sources may also shift in their spectral output. This is most notable in LEDs, which can shift up to a few nanometers, depending on how they are driven. Of course, such a spectral shift will manifest in uncertainties in the effective stimulus and may also introduce undesirable artifacts (such as the unwanted stimulation of photoreceptors). As the direction and size of these spectral shifts cannot be predicted easily, a practical solution is to measure the spectral output at different input settings. These measurements can then be used to design stimuli that account for the spectral shifts or to characterize the uncertainty in stimulus presentation *post-hoc*, or both.

### Temporal Resolution Limits and Time Constants

A historical precedent cautioning to calibrate the temporal output of light generators carefully was offered by Mollon and Polden (24). Measuring the time constants of tachistoscopes, which were devices enabling very brief light exposures pre-dating the common usage of computer displays in research, they found that the time constants were too slow to present stimuli at the millisecond scale accurately. More than 40 years later, accuracy of timing is still a topic in visual psychophysics, with each generation of novel display technology bringing its own potential idiosyncratic temporal artifacts (25–29).

## Physiological Relevance of Time-Varying Stimuli

### Temporal Properties of Distal vs. Proximal Stimuli

In the previous discussion, the near-exclusive focus has been on accurately capturing the temporal properties of the stimulus in the physical domain. In psychophysics, this is sometimes called the distal stimulus, while the pattern of light impinging on the retina is called the proximal stimulus. Notably, the distal stimulus and the proximal stimulus are related to one another through the optics of image formation and projection onto the retina, but they are not the same. Before light excites the photoreceptors, it is modified (relative to the cornea) by passing through the pupil and the ocular media.

#### Pupil Size

Pupil size, being the aperture of the eye, changes the overall retinal irradiance over a factor of ~16 × , or 1.2 log units, given by the ratio of the largest possible pupil area under full dark adaptation (8 mm) to the smallest possible pupil area under bright light conditions (2 mm) (30). The pupil is not static and responds to light in a wavelength- and time-specific fashion. Under time-varying stimuli, then, the pupil size is also dynamically changing, modifying the temporal properties of the distal stimulus in a way that is not very easy to predict. In addition, the pupil responds to other factors unrelated to light, such as cognitive processing (31), and displays spontaneous fluctuations (32, 33) which have been found to be related sleepiness (34). Furthermore, pupil size varies with age, with older people having smaller pupils on average (35), and is subject to diurnal variations (36–41).

As expected, when pupil size is controlled through pharmacological dilation and the stimulus is viewed through this maximally dilated pupil, the same corneal irradiance leads to more melatonin suppression compared with the undilated pupil (42). As a consequence, dose–response curves collected under undilated conditions [e.g., (43)] represent a mixture of two effects: a pupil size effect that modifies retinal light exposure, and a melatonin-suppressive effect. Special care must be taken when dose–response curves collected under different pupil conditions are compared (1, 44).

One solution to estimating retinal illuminance may be the use of mobile eye trackers during a given experiment [e.g., (45)], which enable the determination of pupil size at all time points. In conjunction with head-referenced irradiance or radiance measurements of the corneal irradiance, capturing an individual's “spectral diet” (46), the time course of light exposure at the cornea, it is then possible to determine the actual retinal irradiance, as this is the biologically relevant quantity. Development efforts for such a system are currently underway [e.g., (47)].

#### Eye Movements

Of course, observers are moving their trunk, head, and eyes during the waking day, thereby displacing the retinal image at a high frequency (48). Saccadic eye movements displace the retinal image (49), thereby repositioning different parts of the visual world into the fovea, thereby supporting various visually guided tasks and support various tasks (50, 51). Saccadic eye movements are guided by a variety of factors, including salience and higher-level factors (52). In free-viewing of pictures, saccades can make up to 20% of time (53). In addition, during periods of fixation, three types of fixational eye movements occur: tremor, also called physiological nystagmus, occurring at ~90 Hz, drift, and microsaccades, occurring at 1–2 Hz (54). The extent to which the trunk, head, and eye movements decorrelate the distal from the proximal (retinal) stimulus depends on the spatial characteristics of the scene. One can imagine two extremes. In a completely unarticulated homogenous environment such as those produced by a ganzfeld stimulus, the retinal stimulus to an observer may be nearly constant. On the other extreme, a point light source in an otherwise dark room under free-viewing conditions will stimulate different retinal locations between and—due to fixational eye movements—during fixation, thereby producing very large spatial and temporal contrast.

### Temporal Integration in Circadian and Neuroendocrine Photoreception

Box 2 **Recommended further reading.**

- The CIE has published two documents on documenting lighting and light exposures: CIE 213:2014 (22) and CIE TN 011:2020 (6). The articles published Spitschan et al. (5) and Knoop et al. (21) represent independent proposal for standardized ways of reporting lighting conditions in experimental situations.
- Watson (73) provides a solid introduction into linear-systems modeling of temporal sensitivity in human psychophysics.
- Kronauer et al. (56) synthesize temporal non-linearities in the circadian system's response to light and subject it to rigorous modeling.
- Schlangen & Price (74) provide an extensive introduction in measuring the lighting environment.
- Münch et al. (75) and Knoop et al. (76) provide recent syntheses on the status of daylight for humans, which may serve as a reference when thinking about naturalistic light exposure and its temporal properties.

Ultimately, the use of temporally patterned stimuli biases processing in the retina to a specific set of photoreceptors. While previous work has examined how different photoreceptors drive the pupillary light reflex in different temporal regimes (55), there are at present no parametric measurements for the effects of photoreceptor-selective stimuli varying in their frequency properties on neuroendocrine and circadian physiology. A few studies (2, 3, 56) address the question of temporal integration, though a lot remains unknown in humans. The growing body of literature in animal models [rodents: (57–60), *Drosophila*: (61, 62)] can serve as a useful starting point.

It may be desirable to derive a summary metric for time-varying stimuli. For pulse stimuli, one intuitive way to summarize illuminance or irradiance and duration is the product of the two. This yields a summary quantity with units in e.g., “lux minutes.” This approach has been used in literature to describe total light doses [e.g., (12–14, 63–65)], in line with the notion that the circadian system acts as a photon counter (66). The extent to which this is a useful metric, however, is not necessarily clear. It relies on reciprocity, which refers to the notion that irradiance and duration can be traded off without any difference in effect. Further data mapping out how intensity and duration can be traded off (67) are required before the adoption of such a summary metric.

In field experiments where both light exposure and an outcome metric (such as sleep timing and duration, or other circadian, neuroendocrine, behavioral, and cognitive outputs) are measured conjointly, summarizing the pattern of light exposure is often a necessary step to relate input to output. A data-driven approach to summarizing light exposures in these contexts is to use *time above threshold* (TAT), as a well as *distributional characteristics* of the light exposure above the threshold, such as the mean and standard deviation (68).

### An Outlook

As the temporal properties of the proximal (retinal) stimulus cannot easily be determined, understanding the properties of the distal stimulus is a key first step. One strategy is to quantitatively characterize the propagation of light in specific study context using simulation software (69). This can be achieved using specialized architectural lighting design tools. An integrated workflow to go from physically realistic simulations of illuminated spaces to physiologically plausible simulations of retinal illumination [such as those available in the iSETBIO toolbox (70–72)] is a promising and exciting path forward. For readers wishing to learn more about the fundamentals touched upon in this review, Box 2 provides advanced reading for readers with a background in signal processing and analyses of time-varying signals but no specific background in chronobiology and sleep research.

## Author Contributions

MS: conceptualization, methodology, software, resources, writing—original draft preparation, writing—review and editing, and visualization.

## Conflict of Interest

The author declares that the research was conducted in the absence of any commercial or financial relationships that could be construed as a potential conflict of interest.
